# Genetic analysis and mapping of dwarf gene without yield penalty in a γ-ray-induced wheat mutant

**DOI:** 10.3389/fpls.2023.1133024

**Published:** 2023-03-22

**Authors:** Qingguo Wang, Hongchun Xiong, Huijun Guo, Linshu Zhao, Yongdun Xie, Jiayu Gu, Shirong Zhao, Yuping Ding, Luxiang Liu

**Affiliations:** ^1^ School of Life Sciences, Qingdao Agricultural University, Qingdao, China; ^2^ National Key Facility for Crop Gene Resources and Genetic Improvement, National Center of Space Mutagenesis for Crop Improvement, Institute of Crop Sciences, Chinese Academy of Agricultural Sciences, Beijing, China

**Keywords:** wheat, plant height, dwarf mutant, QTL, γ-ray

## Abstract

Plant height is one of the most important agronomic traits that affects yield in wheat, owing to that the utilization of dwarf or semi-dwarf genes is closely associated with lodging resistance. In this study, we identified a semi-dwarf mutant, *jg0030*, induced by γ-ray mutagenesis of the wheat variety ‘Jing411’ (wild type). Compared with the ‘Jing411’, plant height of the *jg0030* mutant was reduced by 7%-18% in two years’ field experiments, and the plants showed no changes in yield-related traits. Treatment with gibberellic acid (GA) suggested that *jg0030* is a GA-sensitive mutant. Analysis of the frequency distribution of plant height in 297 F_3_ families derived from crossing *jg0030* with the ‘Jing411’ indicated that the semi-dwarf phenotype is controlled by a major gene. Using the wheat 660K SNP array-based Bulked Segregant Analysis (BSA) and the exome capture sequencing-BSA assay, the dwarf gene was mapped on the long arm of chromosome 2B. We developed a set of KASP markers and mapped the dwarf gene to a region between marker PH1 and PH7. This region encompassed a genetic distance of 55.21 cM, corresponding to a physical distance of 98.3 Mb. The results of our study provide a new genetic resource and linked markers for wheat improvement in molecular breeding programs.

## 1 Introduction

Bread wheat (*Triticum aestivum* L.) is one of the most important food crops in the world. It is urgent to increase grain yield to meet the demands of the expanding human population and to mitigate the effects of climate change. Plant height significantly correlates with lodging resistance and therefore plays an important role in yield stability in wheat (Fan et al., 2012). Identification of novel QTLs or genes for plant height is of great significance for improving grain yield in wheat.

In the 1960s, the dwarfing genes *Rht1*(*Rht-B1b*) and *Rht2*(*Rht-D1b*) were introduced into wheat varieties in breeding, which significantly improved lodging resistance and harvest index, resulting in the well-known ‘Green Revolution’ (Peng et al., 1999; Würschum et al., 2017). Single-base mutations in the nucleotide sequences coding for the DELLA domains in *Rht1* and *Rht2* produced truncated proteins which resulted in the dwarf phenotypes. These genes do not respond to GA treatment and are thus categorized as GA-insensitive dwarfing genes (Hedden, 2003). Generally, the *Rht1*/*Rht2* genes and their allelic variants (*Rht3*, *Rht10*, *Rht11*, *Rht17*) are classified as GA-insensitive. The green revolution genes *Rht1* and *Rht2* derived from the wheat cultivar ‘Norin 10’ are located on the short arms of chromosomes 4B and 4D, respectively. The effect of *Rht1* was to reduce plant height by approximately 20%, and that of *Rht2* was to reduce height ~24% (Miralles and Slafer, 1995; Sial et al., 2002). *Rht1* and *Rht2* also affect heading date, spikelet development, tiller number, and the number of kernels per spike (Würschum et al., 2015; Mo et al., 2018). *Rht3* has a stronger effect on height reduction than either *Rht1* or *Rht2* due to a 2-kb retrotransposon insertion in the coding region of the DELLA protein (Flintham and Gale, 1983; Wen et al., 2013). The presence of two copies of the *Rht2* tandem segmental duplication in *Rht10* causes an extremely dwarf phenotype (Li Y., et al., 2012). *Rht11* and *Rht17* are allelic variations of *Rht1*, which also have premature stop codons present in the DELLA domain (Divashuk et al., 2012; Li A., et al., 2012; Bazhenov et al., 2015).

Along with the refinement of wheat genome sequences (Appels et al., 2018), several GA-sensitive dwarfing genes have been cloned in recent years including *Rht8*, *Rht12*, *Rht13*, *Rht18*, *Rht23*, and *Rht24*. *Rht8*, which is located on the short arm of chromosome 2D, has a frameshift mutation in an RNase H-like protein and regulates plant height through modification of the bioactive GA content (Chai et al., 2022; Xiong et al., 2022). Mutation analysis suggests that the gene encoding GA2oxidaseA13 on chromosome 5A contributes to dwarf phenotype of *Rht12* plants (Buss et al., 2020). *Rht13* is located on the long arm of chromosome 7B (Ellis et al., 2005), and a recent study found that the dwarf phenotype of *Rht13* results from single nucleotide variation in a NB-LRR protein (Borrill et al., 2022). *Rht18* is located on chromosome 6A, and the increased expression of *GA2oxA9* in *Rht18* results in decreased plant height (Ford et al., 2018). *Rht23* is located on the long arm of chromosome 5D, and it has been reported that *5Dq* is a candidate gene for *Rht23* (Chen et al., 2015; Zhao et al., 2018). *Rht24* is located on the long arm of chromosome 6A, and encodes a gibberellin (GA) 2-oxidase; it reduces plant height without affecting yield and also increases photosynthetic rate and nitrogen use efficiency (Tian et al., 2022).

It is important to note that a number of dwarf genes have only been located to the chromosomal regions and have not yet been cloned. *Rht4* is located on the long arm of chromosome 2B; a 36 Mb deletion derived from the dwarf line DD399 was identified in this region (Wu et al., 2021). *Rht5* (Ellis et al., 2005), *Rht6* (Mohan et al., 2021), and *Rht22* (Peng et al., 2011) are located on chromosomes 3BS, 4D, and 7AS, respectively. *Rht7* and *Rht21* are located on 2AS (Lu et al., 2015), while *Rht9* and *Rht12* are located on 5AL (Sutka and Kovács, 1987; Ellis et al., 2005; Sun et al., 2019); *Rht14*, *Rht16* and *Rht25* are all located on 6AS (Mo et al., 2018; Vikhe et al., 2019; Gong et al., 2021).

Although more than 20 dwarf genes have been documented in wheat, only a few genes are presently utilized in wheat breeding. More loci or genes that regulate plant height without influencing yield components need to be investigated. In this study, we identified a semi-dwarf mutant named *jg0030* that shows no yield penalty. BSA and genetic mapping in an F_2_ population derived from crossing *jg0030* with the ‘Jing411’ showed that a QTL located on the long arm of chromosome 2B was responsible for height reduction in the mutant. The identification of the semi-dwarf mutant and the QTL for height regulation provide important resources for the development of wheat varieties with a high degree of yield stability.

## 2 Materials and methods

### 2.1 Plant materials and growth measurement

The semi-dwarf mutant *jg0030* was induced by γ-ray mutagenesis and the wheat variety ‘Jing411’ was the wildtype. The field experiment was carried out at the Beijing Zhongpuchang and Changping Experimental Stations of the Institute of Crop Sciences, Chinese Academy of Agricultural Sciences. We measured 1,000-grain weight, number of kernels per spike, and effective spike number in the ‘Jing411’ and mutant *jg0030* in plants collected from the plots in 2015 and 2017. We also measured plant height in F_1_ plants produced by crossing *jg0030* with the conventional cultivar ‘Nongda5181’ and also with the ‘Jing411’. Plant heights of ‘Jing411’, ‘Nongda5181’, and *jg0030* were measured at maturity in 2017 and 2018. Four segregating F_2_ populations were produced by self-pollinating the Jing411/*jg0030*, *jg0030*/Jing411, Nongda5181/*jg0030*, and *jg0030*/Nongda5181 F_1_ hybrids. The F_3_ families were derived from crossing Jing 411 with *jg0030*, and the plant heights were measured at maturity.

### 2.2 Exogenous GA treatment

The seeds of the two parents were selected and soaked in 1% H_2_O_2_ solution for 12 hours to break dormancy. Fifteen seeds were placed on the germination rack in a light incubator with a 12 h light/dark photoperiod at a temperature of 21°C. After two days of culture with water, 2 mg L^-1^ GA_3_ was added to the experimental group. After 8 days of growth, five seedlings with consistent growth were taken from three replicates to measure seedling length.

### 2.3 DNA extraction and quality determination

Genomic DNA was extracted from ~ 0.1 g of leaf tissue from individual plants. The quality of DNA was assessed *via* electrophoresis on a 1% agarose gel stained with GelRed to observe the integrity of the DNA. The DNA concentration was measured using a NanoDrop One spectrophotometer (Thermo Scientific). The DNA samples were diluted to ~200 ng/uL and stored at -80°C.

### 2.4 BSA using the wheat 660K SNP array

For wheat 660K SNP array-based BSA, 40 extremely tall plants and 40 extremely dwarf plants were selected from the ‘Nongda5181’ × *jg0030* F_2_ segregating population to construct extreme bulks. In addition, 10 individual plants of ‘Nongda5181’ and *jg0030* were used to construct the parental pool, and the mixing pools were constructed by combining equal amounts of DNA from each plant. The Axiom^®^ Wheat 660K SNP Array (Thermo) was used to genotype the two pools and the parental DNA samples. Genotyping was performed by China Gold Marker (Beijing) Biotechnology Co., Ltd. The resulting microarray data were screened and analyzed (Dish QC>0.82; call Rate >94). Finally, mapping of the dwarf gene was performed using the high-quality SNP data obtained from the 660K SNP array.

### 2.5 BSA and exome capture sequencing

The F_3_ families derived from the cross between the ‘Jing411’ and the *jg0030* mutant were sampled. We selected four plants from one tall or dwarf F_3_ line, and a total of 20 F_3_ lines were used for construction of extremely tall or dwarf bulks; two extremely tall or dwarf bulks, and two parental pools containing 10 plants were constructed to exome capture sequencing. Exome capture sequencing mainly includes the following steps; first, the DNA libraries for the extreme bulks and the parental lines were constructed, and the exon probe solution was hybridized with single-stranded DNA. The WheatPanExomeV2 (Tcuni, Chengdu, China) was used as exon probes, which includes 2,574,323 probes covering 137 Mb CDS of Chinese Spring RefSeq V2.1, and also containing specific CDS based on genome sequences from other modern wheat cultivars such as KN9204, AK58 and YZ4110. The uncaptured DNA fragments were then washed away, and the enriched exons were amplified by PCR. After quality control, high-throughput DNA sequencing was performed using the Illumina platform. Filtering the SNPs obtained by mutation detection was performed using the SNP-index algorithm to calculate the genotype frequency of the extreme bulks, and the sites with significant differences between the extreme bulks were statistically screened. The Euclidean Distance (ED) values between the two extreme bulks were calculated by using the depth of each allele, and the candidate region was determined according to the fitting result of the ED.

### 2.6 KASP marker development

According to the exome capture sequencing data, the loci with homozygous differences between the two parents and higher sequencing depth (5<DP<300) were screened. The flanking sequences 100 bp in length on both sides of the loci were downloaded from the wheat genome website IWGSC (http://www.wheatgenome.org/) and the relevant data were submitted to the online primer design website Polymarker (http://polymarker.tgac.ac.uk/) to design genome-specific primers. First, genotype analysis was performed in the two parents to verify the specificity of the markers. These specific markers were then used to identify the genotypes of plants in the F_2_ population. The phenotypic and genotypic data from the F_2_ plants was combined, the linkage relationships between the marker loci and the target gene were analyzed, and the approximate position of the target gene on the chromosome was preliminarily determined.

### 2.7 Linkage map construction and dwarf gene mapping

QTL mapping of the semi-dwarf gene was performed using 288 F_2_ individuals obtained from a cross between the ‘Jing411’ and the *jg0030* mutant. The genotypes of F_2_ population were obtained by KASP assay and the genetic map was constructed by QTL IciMapping4.0. The genetic distance between the linked marker loci was estimated using the Kosambi function based on the recombination frequency.

## 3 Results

### 3.1 Phenotypes and yield-related traits of the semi-dwarf wheat mutant *jg0030*

**Figure 1:**
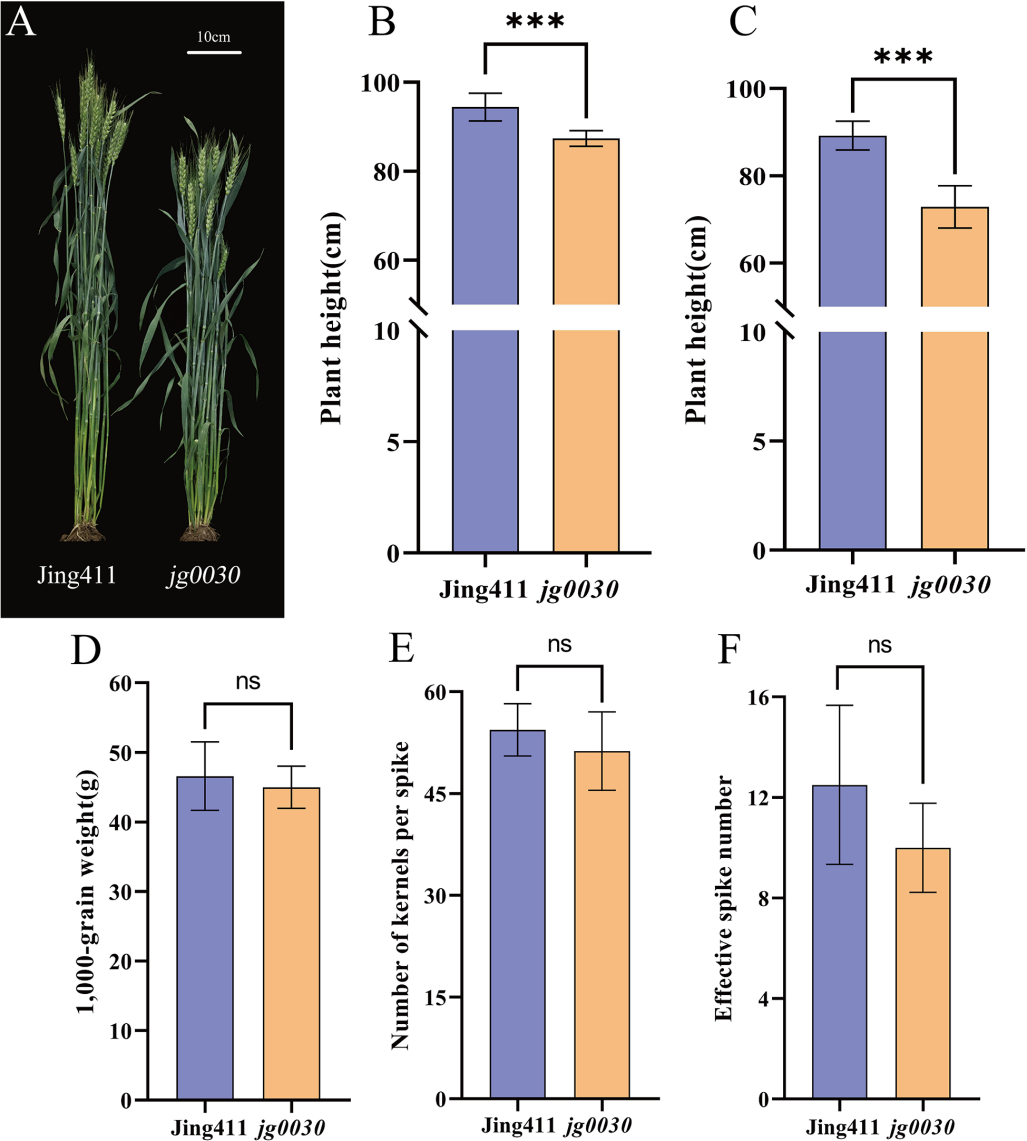
Comparisons of whole-plant phenotypes between the wild type ‘Jing411’ and the dwarf mutant *jg0030*. **(A)** Mature plants of ‘Jing411’ and *jg0030*. Scale bar=10 cm. B-G. statistical analyses of **(B)** mean plant height of ‘Jing411’ and *jg0030* in 2017. **(C)** mean plant height of ‘Jing411’ and *jg0030* in 2018. **(D)** 1000-grain weight in 2017. **(E)** number of kernels per spike in 2017. **(F)** effective spike number in 2017. Bars show the mean SD (n=8). ns p>0.05; *** p<0.001.

From our γ-ray-induced mutant library, we identified a semi-dwarf wheat mutant, *jg0030*, that displayed no negative effects with respect to yield components. Based on the results of field experiments conducted in 2017 and 2018, the average plant height in the ‘Jing411’ was 94 cm (2017) and 89 cm (2018), while plant height in *jg0030* was 87 cm and 73 cm, respectively. Compared with the ‘Jing411’, plant height in the *jg0030* was reduced by 7%-18% (**Figures 1A–C**). For the yield-related traits, there were no significant differences in 1,000-grain weight (**Figure 1D**, **Figures S1A, D**), number of kernels per spike (**Figure 1E**, **Figures S1B, E**), and effective spike number (**Figure 1F**, **Figures S1C, F**) between the ‘Jing411’ and *jg0030*, suggesting that *jg0030* reduced plant height without imposing a yield penalty.

### 3.2 GA sensitivity analysis of the semi-dwarf mutant *jg0030*

**Figure 2:**
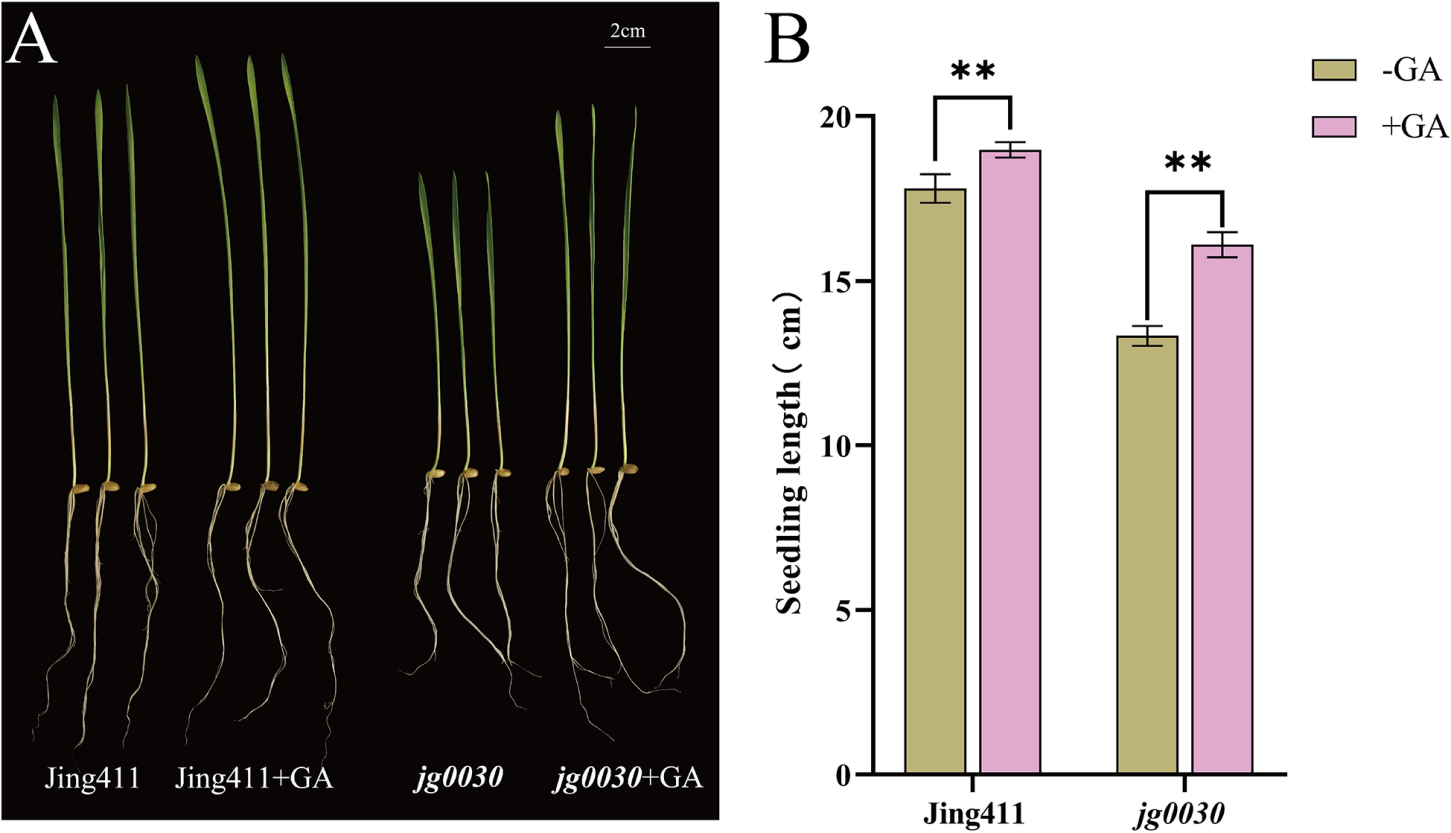
GA sensitivity between the wild type ‘Jing411’ and the dwarf mutant *jg0030*. **(A)** Phenotype of wild type ‘Jing411’ and *jg0030* before and after GA treatment. **(B)** seedling length of ‘Jing411’ and *jg0030* in response to GA treatment. Bars show the mean SD (n=8). **p< 0.01.

In order to clarify the GA sensitivity of the dwarf gene in *jg0030*, exogenous GA treatment was performed on hydroponically grown ‘Jing411’ and *jg0030* seedlings (**Figure 2A**). The results showed that seedling lengths in the ‘Jing411’ and *jg0030* increased by 6.2% and 17.2%, respectively, after exogenous GA treatment (**Figure 2B**), indicating that *jg0030* is a GA-sensitive semi-dwarf mutant.

### 3.3 Genetic analysis of the semi-dwarf gene in *jg0030*

**Figure 3:**
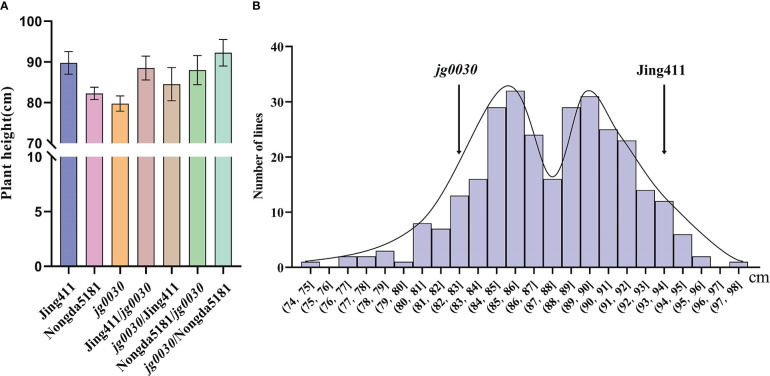
Plant height in F_1_ plants and four F_2_ populations. **(A)** height comparison of the parental lines and F_1_ plants at maturity. Jing411/*jg0030* indicates the F_1_ hybrid in which ‘Jing411’ was the female parent and *jg0030* was the male parent; *jg0030*/Jing411 indicates the F_1_ hybrid in which *jg0030* was the female parent and ‘Jing411’ was the male parent; ‘Nongda5181’/*jg0030* indicates the F_1_ hybrid with ‘Nongda5181’ as the female parent and *jg0030* as the male parent; *jg0030*/Nongda5181 indicates the F_1_ hybrid with *jg0030* as the female parent and ‘Nongda5181’ as the male parent. **(B)** Frequency distributions of plant heights in the F_3_ families derived from crossing Jing411 with *jg0030*.

To analyze the genetic characteristics of the semi-dwarf gene in *jg0030*, we reciprocally crossed the mutant with the ‘Jing411’ and also the relatively tall variety ‘Nongda5181’. The F_1_ plants from all four reciprocal crosses were significantly taller than the *jg0030* plants, and were similar in height to the tall parental lines (**Figure 3A**). These results suggested that the semi-dwarf gene is genetically recessive. We then analyzed the frequency distribution of plant height in 297 F_3_ families derived from crossing Jing411 with *jg0030*, and observed a bimodal distribution (**Figure 3B**), suggesting that the variation of plant height in this population is controlled by a major gene.

### 3.4 Gene mapping using 660K SNP array-based BSA

**Figure 4:**
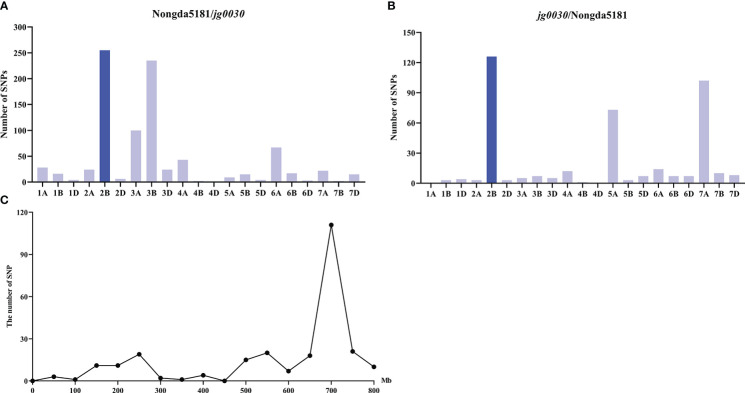
The distribution of SNPs across the 21 T. aestivum chromosomes identified by 660K SNP array-based BSA. **(A)** the Nongda5181/*jg0030* F_2_ population. **(B)** the *jg0030*/Nongda5181 F_2_ population. **(C)** The distribution of SNPs on chromosome 2B.

To investigate the locus/gene that controls the semi-dwarf phenotype in the mutant, we constructed two F_2_ populations from reciprocal crosses of *jg0030* and Nongda5181 consisting of 289 individuals (Nongda5181/*jg0030*), and 304 individuals (*jg0030*/Nongda5181), and selected extremely tall and dwarf F_2_ plants to construct the tall and dwarf bulks for genotyping using the 660K SNP array for BSA. We identified 79,593 SNPs between the two parental lines ‘Nongda5181’ and *jg0030*. After filtration of the SNPs based on the genotypes and phenotypes from the two bulks and the two parental lines, we found that the largest number of SNPs associated with plant height were enriched on chromosome 2B in the two populations, indicating that the gene influencing plant height was located on chromosome 2B (**Figures 4A, B**). We then analyzed the distribution of the associated SNPs on chromosome 2B and identified a large peak between positions 600 and 800 Mb (**Figure 4C**), suggesting that the candidate gene is located on this region.

### 3.5 Gene mapping by exome capture sequencing-BSA assay

**Figure 5:**
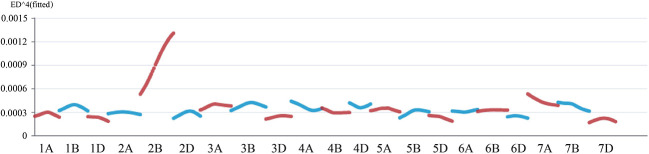
Euclidean Distance correlation analysis by the exome capture sequencing-BSA assay in the Jing411/*jg0030* F_3_ population.

We next selected homozygous dwarf and tall plants in the F_3_ lines derived from the Jing411/*jg0030* populations to construct dwarf and tall bulks and performed exome capture sequencing. According to the genotypes and read depths of the obtained SNPs from the two bulks and the two parental lines, Euclidean Distance (ED) association analysis was performed to determine the linkage strength between each SNP locus and the dwarf gene. Consistent with the results of the 660K SNP array-based BSA, a clear peak was identified at the end of the long arm of chromosome 2B by analysis of the exome capture sequencing data. This result also showed that the gene controlling plant height in the *jg0030* mutant is located on the long arm of chromosome 2B (**Figure 5**).

### 3.6 Validation of the QTL by development of molecular markers

**Figure 6:**
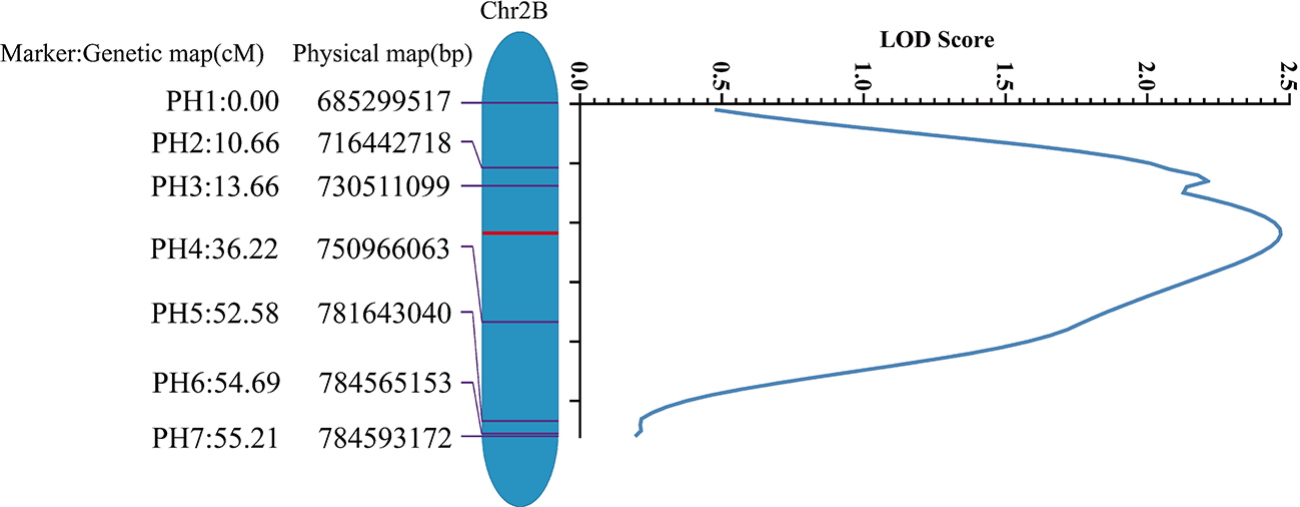
Localization of the detected QTL for dwarf plant stature on the long arm of chromosome 2B in the Jing411/*jg0030* F_2_ population by genetic mapping analysis.

Based on the homozygous SNPs between the ‘Jing411’ and the *jg0030* mutant by exome capture sequencing, we designed 35 KASP markers on the long arm of chromosome 2B. After validation in the two parental lines, a total of seven markers were successfully developed and used to genotype the *jg0030*/Jing411 F_2_ population (**Figures S2A–G**). Based on the genotypes and phenotypes of plants from the *jg0030*/Jing411 F_2_ population, a QTL with a LOD score of 2.5 was detected in a 55.21 cM chromosomal region between marker PH1 and PH7, corresponding to a physical interval of 98.3 Mb (**Figure 6**).

## 4 Discussion

Plant height is one of the important agronomic traits that is closely associated with lodging resistance and is thus important for yield stability in wheat. The utilization of dwarf or semi-dwarf genes is the key component for development of new high-yielding wheat varieties with lodging resistance. At present, the dwarf genes utilized in wheat breeding are mainly *Rht1*, *Rht2*, *Rht8*, *Rht9*, and *Rht24* (Zhong et al., 2018). In addition to reduced plant height, *Rht1* and *Rht2* can increase grain number per spike to improve grain yield (Peng et al., 1999), while *Rht8* (Chai et al., 2022; Xiong et al., 2022) and *Rht24* (Tian et al., 2017) have no negative effects on 1,000-grain weight. In this study, we identified a semi-dwarf wheat mutant, *jg0030*, with high yield potential. Compared with the ‘Jing411’, the height of *jg0030* plants was reduced by 7-18% in two years’ field experiments, and there was no significant change in 1,000-grain weight, grain number per spike, and effective spike number in *jg0030*, demonstrating that the *jg0030* mutation reduces plant height without affecting yield components. Therefore, the dwarf gene in *jg0030* can be used to reduce plant height and thus improve lodging resistance in wheat breeding.

Results of a previous study indicated that the *Rht4* gene is located on the long arm of chromosome 2B and is linked to the SSR marker locus *Xwmc317* (Ellis et al., 2005). Using a dwarf mutant, DD399, a locus for reducing plant height was delimited to a deleted region of 36 Mb (763-799Mb) at the end of the long arm of chromosome 2B, and this locus is thought to be the same as *Rht4* (Wu et al., 2021). In this study, the dwarf gene in the mutant *jg0030* was mapped to the terminus of 2BL using 660K SNP array-based BSA and exome capture sequencing-BSA. By developing seven KASP markers on 2BL, the dwarf gene was mapped between marker PH1 and PH7 that ranged from 685.3 to 784.6 Mb of the physical position on IWGSC RefSeq v2.0, which overlaps with the mapped region of *Rht4*, suggesting that the gene causing the semi-dwarf phenotype in *jg0030* is likely to be the *Rht4* gene. Previous studies have suggested that the original *Rht4* allele derived from the dwarf mutant Burt *ert* 937 significantly decreased 1,000-grain weight (Du et al., 2018; Lu et al., 2022). In contrast, the *jg0030* mutation showed no significant change in 1,000-grain weight, indicating that this mutation induced by γ-ray irradiation is different from that of *Rht4* allele.

It is well documented that *Rht1*/*Rht2* and its homologous genes or allelic variations are insensitive to GA, while the other dwarf genes are sensitive to GA treatment (Peng et al., 1999). GA affects plant growth by promoting cell elongation and cell division (Hedden, 2020). For the GA-sensitive dwarf mutants, degradation of the growth-inhibiting DELLA protein is induced and therefore normal plant height is restored after exogenous application of GA (Hedden and Sponsel, 2015). Several studies have indicated that the GA sensitivity of dwarf genes affects plant growth at the seedling stage, and these genes that differ with respect to GA sensitivity showed distinct effects on coleoptile length in wheat (Tang et al., 2009). Long coleoptiles are beneficial for absorbing enough water from the soil to ensure the early growth of wheat seedlings in the field, allowing them to adapt well to stress (Condon, 2004). A previous study showed that *Rht4* gene is a GA-sensitive dwarf gene, and the spike number, yield per plant, and 1,000-grain weight increased significantly after exogenous GA_3_ treatment of *Rht4* dwarf plants (Lu et al., 2022). Consistently, the length of *jg0030* seedlings increased significantly after exogenous GA treatment, suggesting that *jg0030* is sensitive to GA. Taken together, the results of our study show that the GA-sensitive dwarf gene present in *jg0030* represents a new genetic resource for the development of high-yielding semi-dwarf wheat cultivars with good resistance to lodging.

In conclusion, our study identified an elite semi-dwarf wheat mutant *jg0030* without yield penalty induced by γ-ray irradiation of the cultivar ‘Jing411’. Exogenous GA treatment suggested that *jg0030* was a GA-sensitive semi-dwarf mutant. We mapped the dwarf gene to the terminal region of 2BL using bulked segregant analysis. Further genetic mapping by development of KASP markers in an F_2_ population derived from crossing the ‘Jing411’ with the *jg0030* mutant indicated that the dwarf gene was located on a 55.21 cM interval between marker PH1 and PH7 on 2BL. The results of our study provide important resources and linked molecular markers for breeding wheat varieties with high lodging resistance and good yield stability.

## Data availability statement

The datasets presented in this study can be found in online repositories. The names of the repository/repositories and accession number(s) can be found below: NCBI BioProject (https://www.ncbi.nlm.nih.gov/bioproject/), PRJNA916817.

## Author contributions

LL conceived the project and revised the manuscript. HX designed the experiment, QW performed most of the experiments and data analysis. HG developed the mutant. YX, LZ, JG, SZ, and YD participated in field trials. All authors contributed to the article and approved the submitted version.
