# Supplementary material for: Genetic analysis and mapping of dwarf gene without yield penalty in a γ-ray-induced wheat mutant
Source: Front Plant Sci. 2023 Mar 22;14:1133024. doi: 10.3389/fpls.2023.1133024 (PMC10074482; doi:10.3389/fpls.2023.1133024)
Supplement: Supplementary file 1 [file DataSheet_1.docx]

Supplementary Material

Genetic analysis and mapping of dwarf gene without yield penalty in a γ-ray-induced wheat mutant

Qingguo Wang^1,2, #^, Hongchun Xiong^2, #^, Huijun Guo^2^, Linshu Zhao^2^, Yongdun Xie^2^, Jiayu Gu^2^, Shirong Zhao^2^, Yuping Ding^2^, Luxiang Liu^2,*^

^1^ School of Life Sciences, Qingdao Agricultural University, Qingdao, China

^2^ National Key Facility for Crop Gene Resources and Genetic Improvement, National Center of Space Mutagenesis for Crop Improvement, Institute of Crop Sciences, Chinese Academy of Agricultural Sciences, Beijing, China

^#^ These authors have contributed equally to this work.

**^*^ Correspondence:**Luxiang Liu
liuluxiang@caas.cn

# Supplementary Figures


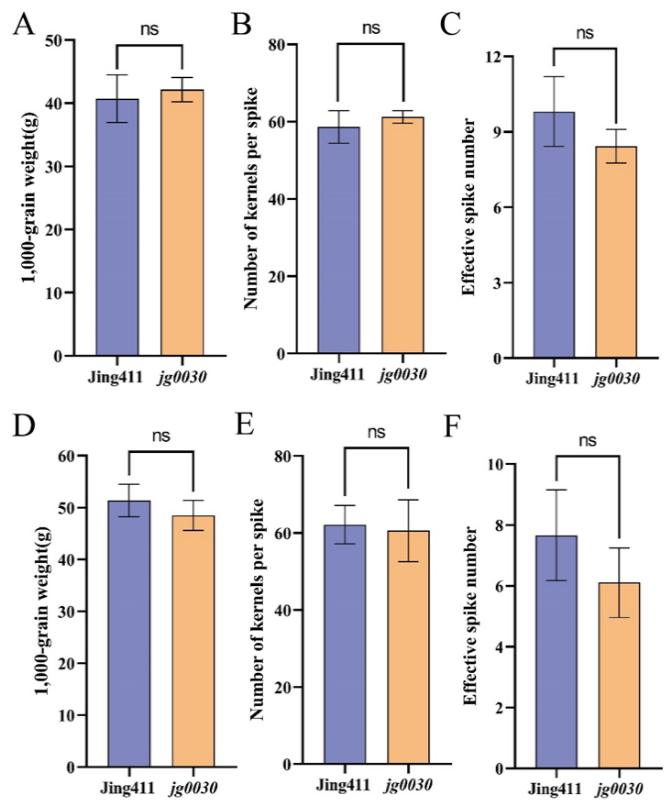


**Supplementary Figure 1.** Comparisons of phenotypes between the wild type ‘Jing411’ and the dwarf mutant *jg0030*. A. 1000-grain weight at Changping experimental stations in 2015. B. Number of kernels per spike at Changping experimental stations in 2015. C. Effective spike number at Changping experimental stations in 2015. D. 1000-grain weight at Zhongpuchang experimental stations in 2015. E. Number of kernels per spike at Zhongpuchang experimental stations in 2015. F. Effective spike number at Zhongpuchang experimental stations in 2015.

**
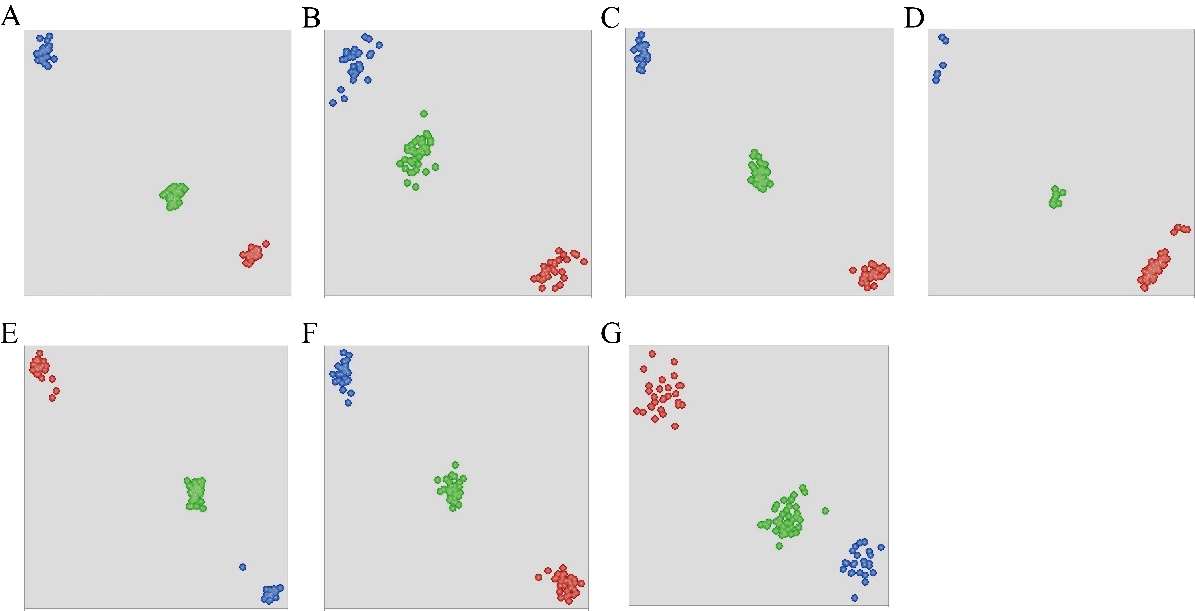
**

**Supplementary Figure 2.** Genotypes detected by KASP markers. A. PH1. B. PH2. C. PH3. D. PH4. E. PH5. F. PH6. F. PH7.
